# Reactive hypoglycemia in binge eating disorder, food addiction, and the comorbid phenotype: unravelling the metabolic drive to disordered eating behaviours

**DOI:** 10.1186/s40337-023-00891-z

**Published:** 2023-09-19

**Authors:** Marianna Rania, Mariarita Caroleo, Elvira Anna Carbone, Marco Ricchio, Maria Chiara Pelle, Isabella Zaffina, Francesca Condoleo, Renato de Filippis, Matteo Aloi, Pasquale De Fazio, Franco Arturi, Cristina Segura-Garcia

**Affiliations:** 1 Psychiatry Unit, Outpatient Unit for Clinical Research and Treatment of Eating Disorders, University Hospital Renato Dulbecco, Catanzaro, Italy; 2 Mental Health Centre of Cirò Marina, Crotone, Italy; 3 Department of Medical and Surgical Sciences, University Magna Graecia of Catanzaro, Catanzaro, Italy; 4 Internal Medicine Unit, Outpatient Unit for the Treatment of Obesity, University Hospital “Renato Dulbecco”, Catanzaro, Italy; 5 Department of Health Sciences, University Magna Graecia of Catanzaro, Catanzaro, Italy; 6 Department of Clinical and Experimental Medicine, University of Messina, Messina, Italy

**Keywords:** Binge-eating disorder, Food addiction, Hypoglycemia, Obesity, OGTT

## Abstract

**Background:**

Impaired metabolic response such as blood glucose fast fluctuations may be hypothesized in binge eating disorder (BED) and food addiction (FA) by virtue of the repetitive consumption of highly processed food. Conversely, rapid changes in plasma glucose (i.e., hypoglycemia) may trigger craving for the same food products. The investigation of early glycemic disturbances in BED and FA could enhance the understanding of the metabolic mechanisms involved in the maintenance of the disorders. Present study investigated hypoglycemia events during a 5-h-long oral glucose tolerance test (OGTT) in people with BED, FA, and the comorbid phenotype. Further, the association between the severity of eating psychopathology and the variability in hypoglycaemia events was explored.

**Methods:**

Two-hundred participants with high weight and no diabetes completed the extended OGTT and were screened for BED, FA, BED-FA, or no-BED/FA. The four groups were compared in hypoglycemia events, OGTT-derived measures, and eating psychopathology. The association between predictors (eating psychopathology), confounders (demographics, metabolic features), and the outcomes (hypoglycemia, early/late hypoglycemia, severe hypoglycemia, reactive hypoglycemia) was examined through logistic regression.

**Results:**

Hypoglycemia in general, and reactive hypoglycemia were highly frequent (79% and 28% of the sample, respectively). Hypoglycemia events (< 70 mg/dL) were equally experienced among groups, whilst severe hypoglycemia (< 54 mg/dL) was more frequent in BED at the late stage of OGTT (5 h; χ^2^ = 1.120, *p* = .011). The FA and BED groups exhibited significantly higher number of reactive hypoglycemia (χ^2^ = 13.898, *p* = .003), in different times by diagnosis (FA: 210′–240′; BED: at the 270′). FA severity was the only predictor of early and reactive hypoglycemia.

**Conclusions:**

People with BED or FA are prone to experiencing reactive hypoglycemia; FA severity may predict early and symptomatic hypoglycemia events. This can further reinforce disordered eating behaviours by promoting addictive responses, both biologically and behaviourally. These results inform professionals dealing with eating disorders about the need to refer patients for metabolic evaluation. On the other hand, clinicians dealing with obesity should screen for and address BED and FA in patients seeking care for weight loss.

**Supplementary Information:**

The online version contains supplementary material available at 10.1186/s40337-023-00891-z.

## Abstract (plain language summary)

Impairment in blood glucose control may be attended in binge eating disorder (BED) and food addiction (FA), two distinct eating disorders which are characterized by the recurrent consumption of highly palatable food rich in high-glucose index carbohydrates. Conversely, rapid changes in blood glucose, such as hypoglycemia, may intensify craving for high-calorie products, thus reinforcing pathological eating behaviours. This study investigated the presence of hypoglycemia events in people suffering from BED, FA, both, or no eating disorder, and explored whether the severity of eating behaviours correlated with a higher probability of having hypoglycemia. Results showed that people with BED and FA experienced more episodes of symptomatic hypoglycemia than those with obesity but no eating disorder. The severity of binge eating was associated with more severe hypoglycemia events, indicated by lower plasma glucose values. Lastly, people with severe FA were more prone to experiencing early post-meal hypoglycemia accompanied by symptoms. These results inform professionals dealing with eating disorders about the need to refer patients for metabolic evaluation. On the other hand, clinicians dealing with obesity should screen for and address BED and FA in patients seeking care for weight loss.

**Supplementary Information:**

The online version contains supplementary material available at 10.1186/s40337-023-00891-z.

## Background

Binge eating disorder (BED) is characterized by recurrent episodes of eating large amount of food in a short amount of time with the subjective feeling of losing control, at least three of the associated features (i.e., high speed, embarrassment while eating, eating when not physically hungry, eating until uncomfortably full, and guilt, or disgust associated with the episodes), and without any compensatory behaviour taking place to counterweigh the food intake [1]. Research that has examined BED from a psychopathological perspective has highlighted that episodes of binge eating are consequential to a state of negative affect, and that the emotional state tends to improve shortly after the binge episode [2, 3].

Food addiction (FA), whom BED has shown to have high co-occurrence and share symptomatology with [4–6], refers to a chronic and relapsing condition characterized by uncontrolled eating in order to achieve pleasure or relieve emotional or physical distress [7, 8]. FA is thought to have neurobiological underpinnings involving the circuitry of reward, and shares clinical and neurobiological similarities with substance use disorders [9, 10].

Beyond that, it is important to acknowledge that food contains various nutrients that could potentially act as triggers of eating behaviours on their own [11]. In FA, the type of food consumed is typically highly processed and contains either macronutrients or pure high-glycemic index carbohydrates [11, 12].

From a metabolic point of view, impaired metabolic response in blood glucose fluctuations may be hypothesized in FA, but also in BED, by virtue of the high-glycemic index carbohydrates content of binge eating episodes [13–15]. As a result, insulin secretion and/or targeted efficacy may alter and fail to maintain euglycemia. Plasma glucose levels play a key role in the short-term daily regulation of the feelings of hunger, satiety, and, therefore, eating habits [16]. It is well known that unfavourable changes in the level of blood glucose and the presence of hypoglycemia episodes may, in turn, promote craving for high-calorie products [14]. Reactive hypoglycemia, defined as plasma glucose ≤ 70 mg/dL (≤ 3.9 mmol/L) within 2–5 h after a meal, is characterized by symptoms such as hands tremor, sweating, palpitations, disorientation, impaired vision, fainting [19]. The hypoglycemia-related stimulation of appetite evoking hormones is then responsible for the drive to eating behaviours [15]. From this perspective, the clinical presentation of reactive hypoglycemia may resemble some of the symptoms which trigger binge-eating episodes and/or FA episodes. On the other hand, recent findings suggest that nearly half of individuals with overweight and obesity experience hypoglycemia without significant symptoms except hunger, and that the frequency of snacking is unrelated to hypoglycemia symptoms. Authors hypothesized that hunger may be induced at a higher threshold than neurogenic symptoms, and that individuals experiencing asymptomatic hypoglycemia might unconsciously prevent symptoms by snacking [20].

De facto, either the characteristics of food consumed, or the behavioural aspect of losing control overeating and craving, raise the question, for BED and FA, if what has traditionally been viewed as a coping mechanism for emotional regulation may have a metabolic counterpart, and that chemical or metabolic mechanisms may be at play, rather than solely neurobehavioral factors [12].

An understanding of the metabolic correlates of BED and FA may aid the general conceptualization of the constructs, including the identification of factors responsible for perpetuating the pathological eating behaviours. Literature on the metabolic correlates of BED revealed a straightforward association with obesity, type 2 diabetes, and metabolic syndrome [21–24]. The research on the metabolic correlates of FA is still in its infancy [25]. Recent studies indicate that FA is common among people with obesity (estimated prevalence ranging from 25 to 42%) [26–28] and is associated with unfavourable outcomes in weight loss management [29]. The prevalence of FA may be even higher in patients undergoing bariatric surgery (57.8%) [30]. While strong support for the association between FA and type 2 diabetes has been recently provided [31], further evidence is required to make definitive conclusions regarding the metabolic-related complications of this disorder [24, 26].

To our knowledge, hypoglycemia, as marker of early onset of glucose metabolism impairment, has not been investigated so far in patients suffering from BED, FA, or in the comorbid phenotype. Present study investigated hypoglycemia events during 5-h-long oral glucose tolerance test (OGTT) in patients with obesity and without diabetes, according to BED, FA, and the comorbid phenotype. It further examined the role of eating psychopathology severity in explaining the variability in hypoglycemia events, when adjusting for demographics, anthropometric, and metabolic features.

## Methods

### Participants

Participants were recruited among people admitted to the diagnostic and therapeutic network of care for obesity (PDTA Obesità) of the University Hospital of Catanzaro (Italy) from May 2017 to December 2022. The PDTA Obesità is a dedicated pathway of care for obesity networked by an interdisciplinary team of specialists (internal medicine, psychiatry, and surgery). Eligibility was met if: (a) men and women aged between 18 and 65 years; (b) body mass index (BMI) ≥ 30 kg/m^2^; (c) consent to execute an extended oral glucose tolerance test (300 min); (d) ability to answer self-reported questionnaires autonomously; and (e) valid informed consent to participate. Conversely, (a) pregnancy, having recently given birth or breast feeding, (b) diagnosis of type 2 diabetes, (c) substance use disorder, and (d) ongoing pharmacological treatment or medical conditions with a clear influence on glucose metabolism (e.g., previous gastrointestinal surgery, peptic ulcer disease, hormonal disorders, known inflammatory diseases, oncological diseases, infectious disorders, corticosteroids, chemotherapy, antipsychotics, mood stabilizers, antidepressants, or biologic drugs) were considered exclusion criteria. Eligible patients were fully informed about the aim and the procedures of the study, and that participation was voluntary and free from any compensation. The study was approved by the institutional review boards (Local Ethical Committee, n. 53/2013), and all the procedures were performed in accordance with the principles of the Declaration of Helsinki [34]. A total of 236 eligible patients agreed to participate. Out of the initial sample, 14 participants consented but did not show up at the psychiatric examination, and 22 did not fulfil psychometric questionnaires (attrition rate 14%). Out of the final sample (N = 200), 20 participants dropped out over time during the oral glucose load (see supplementary material for further details on drop-out analysis).

### Procedures

All the metabolic evaluations were run the first day of the admission to the hospital during the visit with the specialist in internal medicine; information relating to previous/ongoing medical conditions and pharmacotherapy was collected in that occasion. After accounting for the eligibility, patients were asked for participation and addressed to complete the psychiatric evaluation (second visit) within one week.

#### Metabolic evaluation

Anthropometrics, fasting and OGTT-derived plasma glucose and insulin, and dynamic measures of insulin secretion/sensitivity/resistance were extracted for this study. Anthropometric evaluation included height and weight, wearing light indoor clothing and no shoes; then BMI was calculated.

The extended OGTT was performed at 8:00 a.m., after 12-h fasting. Plasma glucose and insulin were tested at baseline, and after 30′, 60′, 90′, 120′, 150′, 180′, 210′, 240′, 270′, and 300′ of oral ingestion of a glucose load containing the equivalent of 75 g anhydrous glucose dissolved in water. During the OGTT, patients were guaranteed a relaxing environment and asked to report any symptoms of malaise. The presence of disabling symptoms or symptoms related to hypoglycemia (e.g., hands tremor, sweating, palpitations, hunger, disorientation, impaired vision, fainting) were followed by glucometer examination. All symptomatic hypoglycemia resulted in discontinuation of the procedure, and a carbohydrate meal was offered. Reactive hypoglycemia was diagnosed by documentation of the Whipple’s triad (i.e., symptoms/signs consistent with hypoglycemia, low plasma glucose concentration, resolution of symptoms with carbohydrates intake) [35]. Hypoglycemia events were registered and then classified by severity (hypoglycemia or severe hypoglycemia for plasma glucose, respectively, ≤ 70 mg/dL (3.9 mmol/L) and ≤ 54 mg/dL (3 mmol/)) [35], time (early or late hypoglycemia occurring, respectively, before and after the 180^th^ minute), and the presence of neuroglycopenic/neurogenic symptoms (i.e., reactive hypoglycemia) for the analysis.

Glucose tolerance profile (GTP) and fasting and OGTT-derived measures of insulin response and sensitivity were calculated from the glucose load. Participants were classified by their glucose tolerance as having normal glucose tolerance (NGT) (fasting plasma glucose < 100 mg/dL and 2 h plasma glucose < 140 mg/dL), impaired fasting glucose (IFG) (fasting plasma glucose 100–125 mg/dl and 2 h plasma glucose < 140 mg/dL), and impaired glucose tolerance (IGT) (fasting plasma glucose < 126 mg/dl and 2 h plasma glucose 140–199 mg/dL) [36]. Early glucose-stimulated insulin response was calculated as the insulinogenic index (Ð plasma insulin (0–30 min)/ Ð plasma glucose (0–30 min) [37]. The homeostatic model assessment insulin resistance index (HOMA-IR) was calculated as fasting plasma insulin (pmol/l) × fasting plasma glucose (mmol/l)/135 [38]. The β cells function and the liver and peripheral tissues sensitivity to insulin were evaluated by Matsuda dynamic index proposed by Matsuda and De Fronzo (10 000/ (fasting plasma insulin × fasting plasma glucose × mean plasma insulin × mean plasma glucose) [39].

#### Psychiatric evaluation

Psychiatrists with adequate training in the field of eating disorders interviewed the participants during the week after the metabolic evaluation. The structured clinical interview for the DSM-5 (SCID-5) [40] was performed to diagnose BED. Given the diagnosis of FA is not formally recognized in the DSM, diagnostic criteria for a probable diagnosis have mainly been extrapolated from the DSM criteria for substance use disorders, as suggested by the common pathogenetic and clinical background for behavioural and drug addictions [41]. The diagnosis of FA was then performed basing on the specific and validated psychometric questionnaire (see below). Participants then completed the following self-reported questionnaires:

- The Binge Eating Scale (BES) is a 16 items questionnaire which assess the severity of behaviours, feelings, and cognitions associated with BED [42–44]. A total BES score < 17 indicates unlikely BED (“mild”), a 17–27 score possible BED (“moderate”) and values > 27 probable BED (“severe”). Cronbach’s alpha in the present study was 0.89.
- The Yale Food Addiction Scale (YFAS 2.0) questionnaire consists of 35 items that assess addiction-like eating behaviours across 11 criteria (e.g., overeating, desire to cut down, time spent, craving, related impairment, risky use, tolerance, and withdrawal) over the past 12 months [45, 46]. The sum of the endorsed symptoms yields a total score (0–11), and a severity level (mild: 2–3 symptoms, moderate:4–5 symptoms, severe > 6 symptoms). It is important to note that the presence of impairment/distress criteria is essential for diagnosing FA, and only the severity level scoring system ensures the presence of this criteria. Thus, the severity level was used for the FA diagnosis confirmation and analysis. Internal consistency (Kuder–Richardson) for this study was 0.86.

According to the presence/absence of the BED/FA diagnosis, the total sample was divided into 4 groups (BED, FA, BED-FA, no-BED/FA).

### Statistical analysis

Dataset manipulation and statistical analyses were performed using the Statistical Package for the Social Sciences for Mac (SPSS; ver. 25). To detect and address for potential outliers within the variables of interest, Mahalanobis' distance and the winsorizing method were employed. After accommodation, skewed variables were identified through Shapiro–Wilk test and natural log-transformed (IGI). Groups were compared in the variables of interest through χ^2^ and multivariate analysis adjusting for potential confounders (BMI, sex, and age), as appropriate. For significant results the effect size (ŋ^2^ values of 0.2, 0.6, 1.2 and > 1.2 considered, respectively, slight, small, moderate, and large effect) [47] and Bonferroni post-hoc analyses were run. Time-to-event analysis (Kaplan–Meier), accounting for censoring, was run to assess the probability for reactive hypoglycemia; log-rank test was used to test significance among groups. Logistic regression served to examine associations between BED/FA severity and the outcomes. Specifically, five regression models were run for the outcomes (hypoglycemia, early/late hypoglycemia, severe hypoglycemia, and reactive hypoglycemia), entering simultaneously predictors (BES severity, YFAS severity), and confounders (age, sex, BMI, GTP, HOMA-IR). To handle type I errors, the Bonferroni correction method was applied within families of tests. Drop-out analysis was performed to address for possible attrition bias. Significant coefficients were considered for corrected p values (specific corrected significance levels in tables).

## Results

Sample description is shown in Table 1. Groups showed similar age and BMI. Female were overrepresented in all groups, with higher percentages in the BED and BED-FA groups (χ^2^ = 10.272, *p* = 0.016). Significant differences by diagnostic group emerged in fasting plasma glucose (F = 4.365, *p* = 0.006), fasting plasma insulin (F = 4.296, *p* = 0.007), and HOMA-IR (F = 6.386, p < 0.001), with the BED-FA group scoring the highest value in fasting plasma glucose and insulin and the BED group in fasting plasma insulin and HOMA-IR (Table 1). Differences in metabolic variables were also explained by the effect of age (fasting plasma glucose and insulin, insulinogenic index), BMI (fasting plasma insulin, Matsuda index, HOMA-IR), sex (fasting plasma insulin, HOMA-IR), and the combined effect of diagnosis*sex (fasting plasma glucose and insulin, HOMA-IR) (statistics of the multivariate analysis in Table 1). NGT was more frequent in all groups (with BED scoring the highest prevalence, FA the lowest); IFG was more frequent in BED, IGT in FA, and the combined IFG-IGT in the BED-FA group (χ^2^ = 22.090, *p* = 0.009).

**Table 1:** Comparison between groups in demographics, eating psychopathology and OGTT-derived metabolic variables

			Groups				Statistics			
			no-BED/FA (n = 80)	BED (n = 43)	FA (n = 24)	BED-FA (n = 53)	F/χ^2^	*p*	ŋ^2^	Bonferroni post-hoc
Age^†^			41.1 (11.9)	36.5 (12.6)	43 (11.8)	39.6 (13.4)	1.825	ns		
Sex (f)^‡^			58 (72.5)	39 (90.7)	18 (75)	48 (90.6)	10.272	.016		
BMI^†^			40.7 (7.5)	40.6 (7.6)	42.7 (8.4)	41.7 (7.6)	0.599	ns		
BES							196.296	< .001		
		Mild	80 (100)	–	24 (100)	–				
		Moderate	–	13 (30.2)	–	15 (28.3)				
		Severe	–	30 (69.8)	–	38 (71.7)				
YFAS 2.0							232.503	< .001		
		None	80 (100)	43 (100)	–	–				
		Mild	–	–	7 (29.2)	2 (3.8)				
		Moderate	–	–	10 (41.6)	16 (30.2)				
		Severe	–	–	7 (29.2)	35 (66.0)				
FPG^†a^			90.0 (10.4)	88.1 (12.2)	88.7 (10.0)	91.0 (10.8)	4.365	**.006**	0.096	BED-FA > FA, BED
FPI^†b^			18.4 (8.7)	19.9 (11.3)	17.5 (6.8)	19.3 (10.5)	4.296	**.007**	0.094	BED, BED-FA > OB, FA
Matsuda index^†c^			2.5 (1.2)	3.1 (1.7)	2.4 (1.3)	2.4 (1.3)		ns		
HOMA-IR^†d^			4.1 (2.0)	4.5 (3.2)	3.8 (1.5)	4.4 (2.7)	6.386	** < .001**	0.134	BED, BED-FA > OB, FA
logIGI^†e^			1.5 (0.3)	1.5 (0.4)	1.4 (0.3)	1.4 (0.3)		ns		
GTP^‡^							22.090	**.009**		
	NGT		49 (61.3)	32 (74.4)	11 (45.8)	38 (71.7)				
	IFG		4 (5.0)	5 (11.6)	–	1 (1.9)				
	IGT		18 (22.4)	3 (7.0)	10 (41.7)	6 (11.3)				
	IFG + IGT		9 (11.3)	3 (7.0)	3 (12.5)	8 (15.1)				

Bold; significant *p*-value after Bonferroni correction for multiple comparisons (set at p < .01)

BED: binge eating disorder; FA: food addiction; BMI: body mass index; BES: binge eating scale; YFAS: yale food addiction scale; FPG: fasting plasma glucose; FPI: fasting plasma insulin; HOMA-IR: homeostasis model assessment insulin resistance; IGI: insulinogenic index; GTP: glucose tolerance phenotype; NGT: normal glucose tolerance; IFG: impaired fasting glucose; IGT: impaired glucose tolerance

Insulinogenic index (IGI) was log-transformed before analysis. ^†^ Means and standard deviations; ^‡^ Frequencies and percentages. Model adjusted for diagnosis, BMI, sex, and age

^a^Significant effect of diagnosis (F = 4.365; *p* = .006; ŋ^2^ = 0.096), age (F = 11.961; *p* = .001; ŋ^2^ = 0.088), diagnosis*sex (F = 3.879; *p* = .011; ŋ^2^ = 0.086)

^b^Significant effect of diagnosis (F = 4.269; *p* = .007; ŋ^2^ = 0.094), BMI (F = 15.319; *p* =  < .001; ŋ^2^ = 0.110), sex (F = 13.521; *p* = .001; ŋ^2^ = 0.098), age (F = 8.935; *p* = .003; ŋ^2^ = 0.067), diagnosis*sex (F = 4.649; *p* = .004; ŋ^2^ = 0.101)

^c^Significant effect of BMI (F = 14.452; *p* =  < .001; ŋ^2^ = 0.104)

^d^Significant effect of diagnosis (F = 6.386; p < .001; ŋ^2^ = 0.134), BMI (F = 14.432; p < .001; ŋ^2^ = 0.104), sex (F = 9.476; *p* = .003; ŋ^2^ = 0.071), diagnosis*sex (F = 6.486; p < .001; ŋ^2^ = 0.136)

^e^Significant effect of age (F = 18.542; p < .001; ŋ^2^ = 0.130)

Table 2 shows frequencies and percentages of hypoglycemia events. A flow-chart describing the interruptions of the OGTT due to hypoglycemia events or drop-out is embed within the supplementary material (Additional file 1: Figure S1). Drop-out analysis revealed no effect of drop-out on the variables of interest (Additional file 1: Table S1). A total of 158 participants (79%) experienced hypoglycemia events during the OGTT, with the highest incidence in the FA group (87.5%, *p* = ns). Hypoglycemia was significantly more frequent in the no BED/FA and BED-FA group at the 270′ (χ^2^ = 10.732, *p* = 0.013), although significance lost after multiple comparison correction (p < 0.008). Severe hypoglycemia (plasma glucose < 54 mg/dL) was more frequently endorsed in the late stages of the glucose load, with the BED group experiencing severe hypoglycemia at the 300′ (χ^2^ = 1.120, *p* = 0.011).

**Table 2:** Hypoglycemia events

		Groups				Statistics	
		no-BED/FA (n = 80)	BED (n = 43)	FA (n = 24)	BED-FA (n = 53)	χ^2^	*p*
Hypoglycemia (< 70 mg/dL)							
Events (yes)		64 (80.8)	30 (69.8)	21 (87.5)	43 (81.1)		ns
Repeated events		57 (71.3)	25 (58.1)	15 (62.5)	38 (71.7)		ns
By OGTT	30′–120′	5 (6.3)	7 (16.9)	1 (4.2)	5 (9.4)		ns
	150′	7 (8.8)	1 (2.4)	2 (8.3)	5 (9.4)		ns
	180′	19 (24.7)	5 (12.8)	8 (34.8)	16 (32.7)		ns
	210′	38 (52.8)	16 (44.4)	13 (59.1)	26 (57.1)		ns
	240′	41 (62.1)	21 (60.0)	12 (63.2)	30 (68.2)		ns
	270′	42 (71.2)	11 (39.3)	5 (41.7)	27 (67.5)	10.732	.013
	300′	25 (48.1)	6 (27.3)	5 (50.0)	17 (42.5)		ns
	Early (200)	25 (31.3)	11 (25.6)	10 (41.7)	19 (35.8)		ns
	Late (175)	59 (81.9)	27 (75.0)	19 (86.4)	40 (88.9)		ns
Severe hypoglycemia (< 54 mg/dL)							
Events (yes)		17 (21.3)	11 (25.6)	7 (29.2)	17 (32.1)		ns
By time	30′–120′	–	–	–	–		
	150′	1 (1.3)	–	–	1 (1.9)		ns
	180′	3 (3.8)	2 (4.7)	–	4 (7.5)		ns
	210′	4 (5.0)	2 (4.7)	4 (16.7)	6 (11.3)		ns
	240′	11 (13.8)	6 (14.0)	4 (16.7)	9 (17.0)		ns
	270′	3 (3.8)	1 (2.3)	2 (8.3)	2 (3.8)		ns
	300′	–	3 (7.0)	–	–	11.120	.011
Reactive hypoglycemia							
Events (yes)		20 (25.1)	15 (34.9)	13 (54.3)	8 (15.1)	13.898	**.003**
By time	30′–120′	–	–	–	–		
	150′	–	2 (4.6)	–	–		ns
	180′	3 (3.7)	–	1 (4.2)	3 (5.7)		ns
	210′	5 (6.3)	–	4 (16.7)	1 (1.9)	10.485	.015
	240′	6 (7.5)	7 (16.3)	7 (29.2)	4 (7.5)	9.972	.019
	270′	6 (7.5)	6 (14.0)	1 (4.2)	–	7.962	.047
	300′	–	–	–	–		

Bold: significant *p*-value after Bonferroni correction for multiple comparisons (set at p < .008)

BED: binge eating disorder; FA: food addiction. Severe hypoglycemia was considered for plasma glucose <  = 54 mg/dl. Early hypoglycemia: 0′–180′; late hypoglycemia > 180′. Sample size for hypoglycemia by OGTT timing due to interruption as follows: 150′ (N = 188), 180′ (N = 174), 210′ (N = 162), 240′ (N = 137), 270′ (N = 124), 300′ (N = 124), early (N = 200), late (N = 175)

^†^Means and standard deviations

**Fig. 1:**
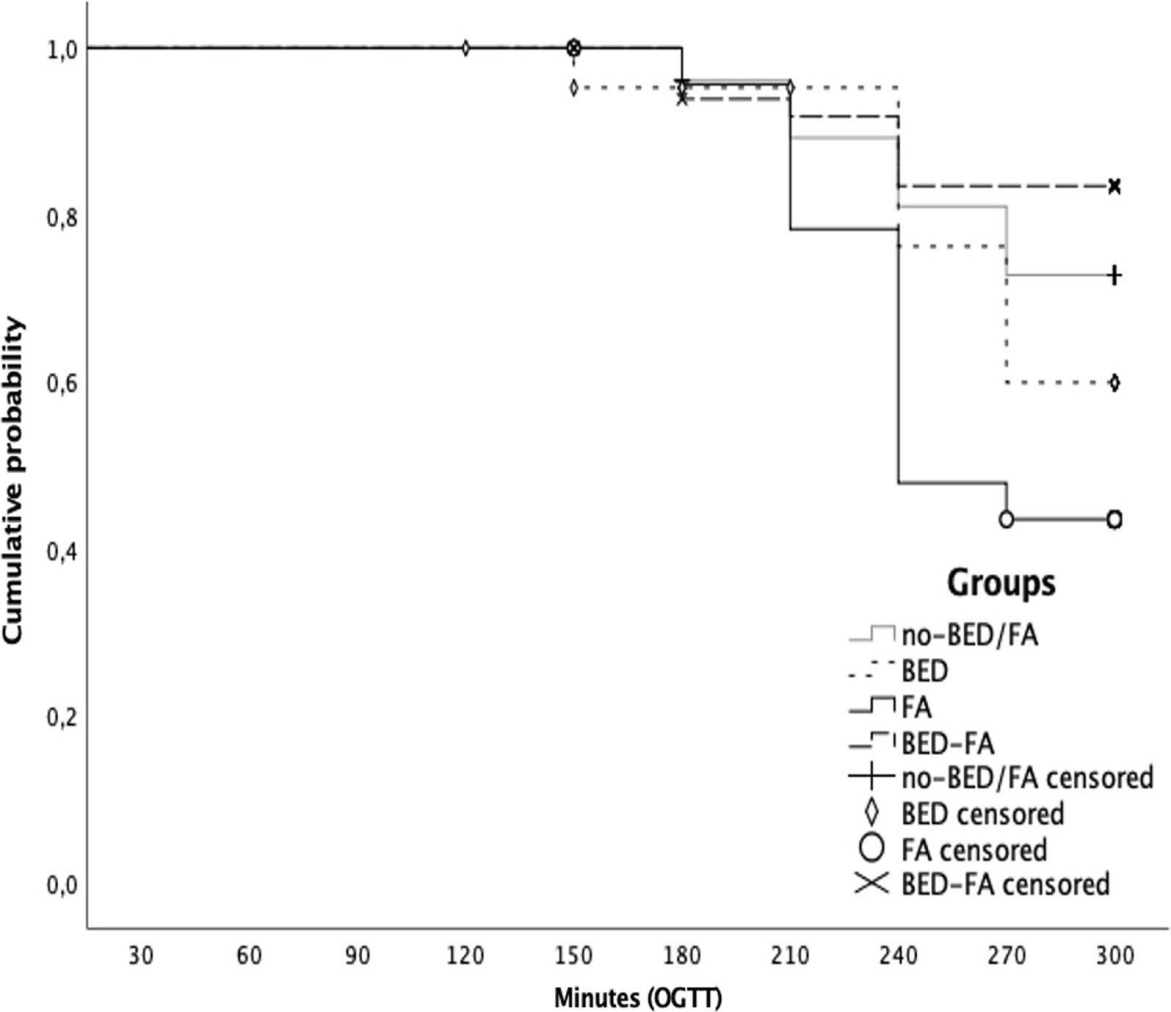
Reactive hypoglycemia and OGTT interruption: time-to-event analysis

Reactive hypoglycemia was documented in 28% (N = 56) of the sample, globally considered. By groups, the FA and BED groups experienced significantly higher number of episodes compared to other groups (χ^2^ = 13.898, *p* = 0.003; FA, BED > no-BED/FA, BED-FA); by time, reactive hypoglycemia was more frequently experienced between the 210′–240′ in the FA group, and at the 270′ in the BED group (significance lost after multiple comparison correction). Time-to-event analysis showed significant lower time to reactive hypoglycemia in the group with FA with respect to the other groups (Mantel Cox Log-Rank test; χ^2^ = 13.952, *p* = 0.003; Fig. 1).

Results from the logistic regression analysis are shown in Table 3. When entering all the variables, no predictors associated with hypoglycemia events, and late hypoglycemia. Early hypoglycemia was significantly associated with severe FA, with participants suffering from severe FA experiencing 14 times more likely early hypoglycemia (*p* = 0.001). Severe hypoglycemia was significantly associated with BMI, although significance was beyond the set corrected *p*-value (0.007). Lastly, FA severity was associated with reactive hypoglycemia, although this associations did not remain significant after Bonferroni correction (0.008 vs 0.007).

**Table 3:** Results of logistic regression for hypoglycemia outcomes

Outcome	Predictor		p	Exp(B)	95% CI	
Hypoglycemia^a^	Constant		.633	2.395		
Early hypoglycemia^b^	FA severity	Severe	.**001**	14.013	2.933	66.953
	Constant		.187	.053		
Late hypoglycemia^c^	Sex (f)		.033	2.992	1.092	7.818
	Constant		.699	.467		
Severe hypoglycemia^d^	BMI		.038	0.940	.887	.997
	Constant		.492	3.572		
Reactive hypoglycemia^e^	FA severity	moderate	.008	14.562	2.031	104.412
	Constant		.519	.294		

Bold: significant *p*-value after Bonferroni correction for multiple comparisons (set at p < .007)

FA: food addiction; BED: binge eating disorder; BMI: body mass index; GTP: glucose tolerance phenotype; NGT: normal glucose tolerance; IFG: impaired fasting glucose; IGT: impaired glucose tolerance; HOMA-IR: homeostasis model assessment insulin resistance. Model adjusted for FA severity, BE severity, BMI, age, sex, GTP, HOMA-IR

^a^Log-likelihood = 150.016, Cox-Snell R^2^ = .081, Nagelkerke R^2^ = .131

^b^Log-likelihood = 147.595, Cox-Snell R^2^ = .173, Nagelkerke R^2^ = .259

^c^Log-likelihood = 157.455, Cox-Snell R^2^ = .104, Nagelkerke R^2^ = .152

^d^Log-likelihood = 185.263, Cox-Snell R^2^ = .086, Nagelkerke R^2^ = .123

^e^Log-likelihood = 183.609, Cox-Snell R^2^ = .114, Nagelkerke R^2^ = .163

## Discussion

Present study investigated hypoglycemia events during extended OGTT in people seeking care for weight loss and suffering from BED, FA, and the comorbid phenotype. It further aimed to explore the extent to which the severity of eating psychopathology accounts for the variability in hypoglycemia events, when adjusting for demographics, anthropometrics, and metabolic features as confounders.

Hypoglycemia is defined as a fall in plasma glucose level beyond the minimum considered of 70 mg/dL (3.8 mmol) [19]. Reactive hypoglycemia, more specifically, refers to hypoglycemia events associated with neurogenic or neuroglycopenic symptoms, which typically revert with carbohydrates intake [48]. Although reactive hypoglycemia may not associate with metabolic disturbances in people with normal weight [49], some evidence suggests it associates with glucose metabolism impairment in its early stage, and, consequently, with increased risk of late diabetes [50, 51]. On the other hand, it is reported that patients suffering from obesity exhibit higher rate of reactive hypoglycemia after oral glucose load than normal weight [52], suggesting that this population is further exposed to develop metabolic complications.

Hypoglycemia, globally considered, was detected in up to 80% of participants. Reactive hypoglycemia, on the other hand, was experienced from 15 to 54% of the sample. These frequencies were unexpectedly high, especially considering that participants had no diabetes and were not exposed to any medical condition or drug affecting glucose metabolism. One study previously investigated reactive hypoglycemia after 4-h long OGTT according to different BMI, founding a prevalence up to 45% in patients with obesity [52]. This result should be read considering that Xue and colleagues adopted a very stringent cut-off (< 55 mg/dL) for the hypoglycemia event, accounting for a smaller prevalence. Some evidence states that autonomic symptoms of hypoglycemia usually occur for plasma glucose < 58 mg/dL [53], and that a strict cut-off is of clinical interest for outcome implications [54]. However, we opted for a more conservative cut-off considering that reactive hypoglycemia related symptoms may present with higher glucose values under the minimum considered of 70 mg/dL, agreeing with other studies on hypoglycemia and other medical conditions [49]. We further considered that intermediate glucose levels (50–70 mg/dL) still have potential to stimulate hunger, with evidence suggesting an association between subthreshold hypoglycemia and the frequency of snacking in daily life [20]. Another factor that may have accounted for the higher reactive hypoglycemia rates, is that the present investigation was conducted on the extended OGTT, which is double the time of a regular oral glucose load (2 h), and one hour longer than Xue and colleagues evaluated [57].

Hypoglycemia, independent of the severity or related symptoms, was more frequent in the late stages of the glucose load for all groups, confirming previous findings of more frequent hypoglycemia at the 4^th^ hour in patients with obesity [52]. This trend was also confirmed considering reactive hypoglycemia, where eating psychopathology did show an effect. To this extent, patients suffering from only FA and BED reported significantly higher frequency of reactive episodes than the other groups, in different times (between the 210′–240′, and at the 270′, respectively). BED was also associated with more severe events at the same timing, although results were not significant after correction for multiple comparisons.

Different factors may account for these results. First, a plausible effect of insulin resistance and hyperinsulinemia should be considered. De facto, all groups exhibited HOMA-IR values suggestive of insulin resistance [58]. The group with BED exhibited the highest plasma insulin and insulin resistance among the groups, confirming previous findings [59–62]. Nevertheless, this mechanism alone does not fully elucidate why the group with FA, whilst reporting the lowest plasma insulin and insulin resistance, still exhibits higher frequencies of reactive hypoglycemia with respect to the other groups. To date, evidence for FA is still scarce and heterogeneous in supporting the association with early disruption in glycemia control [55]. Alternatively, disrupted first phase in insulin secretion has been proposed as a plausible mechanism underlying post-prandial hypoglycemia [66]. According to these hypotheses, low insulin secretion in the first phase would result in high plasma glucose, leading to late and excessive second-phase insulin secretion. We are not able to evaluate if disturbances in the early/late insulin response may play a role, but relatively high and sustained insulin secretion might explain delayed hypoglycemia in FA and BED. An excess of the incretin effect should also be considered [67]. Beyond the effect on insulin secretion in response to glucose ingestion, the glucagon-like peptide-1 (GLP-1) is also responsible for suppressing glucagon response and has been associated with reactive hypoglycemia [68]. Four studies evaluated GLP-1 in binge eating and food addiction, with null findings [69–72]. On the other hand, some promising results emerged from interventional studies using the GLP-1 agonists for the treatment of binge eating and uncontrolled eating, suggesting a positive modulation of craving and food related reward activation [73]. When discussing hormones that are known to contribute to glucose homeostasis and food intake modulation, some other candidates have been recently studied in disordered eating behaviours, binge eating, and food addiction (e.g., ghrelin, leptin, nesfatin-1) [73, 74]. Their potential impact on the correlation between disordered eating behaviours and reactive hypoglycemia should be considered and investigated.

Finally, a recent study evaluated reactive hypoglycemia, snacking habits, and obesity in relation to the index of glucose effectiveness (i.e., the ability to increase peripheral glucose uptake independent of insulin) [75]. Results from this study suggested that either low or high glucose effectiveness relates to hypoglycemia; in the first case, hypoglycemia would result from post-prandial hyperglycemia and hyperinsulinemia (insulin-dependent hypoglycemia). In the second case, high glucose effectiveness would facilitate glucose disposal, predisposing to insulin-independent hypoglycemia. Present results support hyperinsulinemia, insulin resistance, and more severe hypoglycemia at 5 h in BED. On the other hand, patients with FA exhibited higher reactive hypoglycemia frequency, and the severity of FA was associated with early hypoglycemia. Different glucose effectiveness in these two groups could explain the diverse hypoglycemia profiles (insulin-dependent for BED, insulin-independent for FA); however, data about complete insulin load were not available for this analysis, preventing from arguing if this is the case for FA and BED population. By adding new insight on one more hypothesis below the association between FA, BED, and hypoglycemia, Kishimoto and Ohashi also found increased odds of snaking habits according to the lowest and highest percentiles of glucose effectiveness [75].

This is particularly important when dealing with the hypothesis that metabolic profile, hereby in the construct of hypoglycemia, should be considered a risk factor for maintaining the disordered eating behaviours. Previous findings documented the strength to which hypoglycemia and glucose fluctuations promote the motivation for high dense and caloric foods, by modulating limbic/striatal brain regions [76] and triggering addictive responses, biologically and behaviourally [12]. There is also some evidence that insulin interacts with dopamine signalling in the brain areas of reward [77]. Conversely, the consumption of high glycemic index foods is associated with a further surge in glucose and insulin secretion, which may promote subsequent hypoglycemia [78]. Therefore, based on our findings, we could speculate that reactive hypoglycemia, supported by glucose and insulin fluctuations, may reinforce food intake and disordered eating behaviours (e.g., binge eating, food addiction) which in turn may exacerbate reactive hypoglycemia. This is of pivotal importance for both BED and FA, but mostly for the last one, where the craving and addiction-like behavioural profile is more pronounced [6].

It is important to mention that 65% experienced hypoglycemia without reporting related symptoms. The occurrence of hypoglycemia-like symptoms answers to individual and biological factors (e.g., decrease sympathetic response) [79]. Interestingly, there is consensus about autonomic symptoms activating for plasma glucose around 50 mg/dL, while hunger input starts within higher plasma glucose concentrations. Recent evidence found that subclinical reactive hypoglycemia associates with dysfunctional eating behaviours independent of the Whipple’s triad, arguing that “snacking begets snacking through subclinical hypoglycemia” [20]. This might be the case for BED, whom hypoglycemia events were less frequently accompanied by symptoms with respect to patients with FA. Notwithstanding the potentiality of such results, the research scenario on subclinical reactive hypoglycemia is still in its infancy, preventing any direct application in real-word clinical settings. To date, the evaluation and management of hypoglycemia are recommended exclusively for patients meeting the Whipple’s triad criteria (thus, symptomatic) [35], and the definition of reactive hypoglycemia itself does not include asymptomatic episodes [67]. Further evidence is essential to comprehensively assess the long-term clinical impact of subclinical reactive hypoglycemia before revising the concept and translating subclinical reactive hypoglycemia research into clinical practice.

Lastly, studies evaluating the co-occurrence of BED and FA have suggested the this phenotype may represent a more severe form of BED [26], and is associated with worse eating and general psychopathology with respect to simple obesity [80, 81]. In the present study, the comorbid phenotype did not exhibit more hypoglycemia or reactive hypoglycemia events. On the other hand, this group exhibited higher levels of fasting plasma glucose compared to the other groups, higher levels of fasting plasma insulin compared to all groups except those with BED alone, and a more frequent occurrence of impaired fasting glucose plus impaired glucose tolerance (pre-diabetes). It is plausible that hypoglycemia occurs less frequently in this group due to the progression to a later stage of the disorder and a more disrupted glycemic homeostasis. Nonetheless, further investigation is imperative to thoroughly explore and substantiate this hypothesis.

To the best of our knowledge, this is the first evidence so far on the evaluation and characterization of hypoglycemia in people suffering from obesity, without diabetes, and according to BED and FA.

However, present results should be interpreted cautiously considering some limitations. First, the cross-sectional design prevents to evaluate causal relationships between eating psychopathology and hypoglycemia events. Longitudinal studies or intervention studies would provide more robust evidence. Second, the study examined hypoglycemia events during a single testing session which may not capture the full range of metabolic responses and fluctuations in the real-world setting. Although no gold standard is actually recognised for the diagnosis of reactive hypoglycemia [67], and the OGTT has been the most widely used methodology in this field, we acknowledge it is associated with the over-estimation of hypoglycemia incidence [82], preventing any critical translation of results to clinical practice. Real time assessment of glucose variability through continuous glucose monitoring, together with the record of daily nutritional intake, could provide a more comprehensive understanding of the metabolic dynamics and expand actual knowledge about the influence that eating disorders and disordered eating behaviours exert on the metabolic profile. Third, the lack of a healthy normal-weight control group may hinder the ability to compare the metabolic response and hypoglycemia events specifically associated with these conditions. Including healthy subjects with normal weight would allow for better differentiation and interpretation of the results. Finally, while the very strong clinical and biological evidence supports the existence of FA as a distinct clinical phenomenon to be taken into consideration for its long-term complications, FA is not yet incorporated in the current diagnostic systems (i.e., DSM-5 or ICD-11), and lacks well-validated diagnostic criteria. Within this study, the diagnosis of FA relied on the results of the YFAS-2.0 questionnaire, consistent with the literature so far conducted on the topic. Although a recent debate consensus has emerged in support of the Yale Food Addiction Scale as a valid tool to evaluate addictive-like eating behaviors [83], present results certainly require contextualizing the diagnostic constraints.

On the other, it should be considered that the extended OGTT is not formally included in the diagnostic protocol for the diagnosis and treatment of obesity. Accordingly, the major strength of present study is the sample size consistent with the relative low application of the procedure. Further, the sample included male participants, which are usually underrepresented in study on eating disorders.

## Conclusions

Taken together, present results suggest that the frequency of reactive hypoglycemia is higher in patients suffering from obesity and BED or FA and that the severity of food addiction influences early and symptomatic events, independent of metabolic, anthropometric, and demographic features. Hypothetical underlying mechanisms do not fit in explaining this phenomenon in the two groups, needing further investigations on the metabolic biomarkers associated with the glucose homeostasis. At the same time, our findings add to the understanding of the complex relationship between eating psychopathology, metabolic response, and addictive processes.

BED and FA are often associated with obesity, type 2 diabetes, and metabolic syndrome. From the perspective of clinician dealing with eating disorders, these results inform about the need to investigate hypoglycemia and hypoglycemia-related symptoms within the medical assessment. Hypoglycemia may represent an early risk factor for future metabolic complications and need to be assessed and addressed in the specific setting. Moreover, hypoglycemia has the potential to maintain disordered eating behaviours, affecting treatment outcomes.

On the other hand, eating disorders associated with obesity are often underdiagnosed and not addressed in obesity care settings, partly explaining failure of obesity interventions. BED and FA deserve a precise identification and need to be addressed to tailor nutritional and pharmacological treatments. For prevention, early detection, and tailored treatment, a multidisciplinary approach is then necessary.

### Supplementary Information

**Additional file1: Figure S1**. Flow chart describing participants interruption of the glucose load for the events reactive hypoglycemia and attrition. **Table S1**. Comparison between participants completing the OGTT and drop-outs at various level of the glucose load.

## Abbreviations

BED: Binge eating disorder
FA: Food addiction
OGTT: Oral glucose tolerance test
BMI: Body mass index
GTP: Glucose tolerance profile
NGT: Normal glucose tolerance
IFG: Impaired fasting glucose
IFG: Impaired glucose tolerance
HOMA-IR: Homeostatic model assessment insulin resistance index
IGI: Insulinogenic index
DSM: Diagnostic and statistical manual of mental disorders
BES: Binge eating scale
YFAS: Yale food addiction scale

## Declarations

### Availability of data and materials

The datasets used and/or analysed during the current study are available from the corresponding author on reasonable request.
