# Supplementary material for: Reactive hypoglycemia in binge eating disorder, food addiction, and the comorbid phenotype: unravelling the metabolic drive to disordered eating behaviours
Source: J Eat Disord. 2023 Sep 19;11:162. doi: 10.1186/s40337-023-00891-z (PMC10507855; doi:10.1186/s40337-023-00891-z)
Supplement: Supplementary file 1 — Additional file1: Figure S1. Flow chart describing participants interruption of the glucose load for the events reactive hypoglycemia and attrition. Table S1. Comparison between participants completing the OGTT and drop-outs at various level of the glucose load. [file 40337_2023_891_MOESM1_ESM.docx]

Supplementary Material

Figure S1. Flow chart describing participants interruption of the glucose load for the events reactive hypoglycemia and attrition.

Table S1. Comparison between participants completing the OGTT and drop-outs at various level of the glucose load.

|  | | | Groups | | | Statistics | |
| --- | --- | --- | --- | --- | --- | --- | --- |
|  | | | No-drop out  (n=180) | Drop out  (n=20) |  | F/ χ^2^ | p |
| Age^†^ | | | 39.8 (12.2) | 40.9 (15.3) |  | 3.627 | ns |
| Sex (f) ^‡^ | | | 145 (80.6) | 18 (90.0) |  | 1.065 | ns |
| BMI^†^ | | | 41.2 (7.7) | 40.6 (7.4) |  | 0.207 | ns |
|  | | |  |  |  |  |  |
| BES |  | |  |  |  | 6.000 | .05 |
|  | mild | | 94 (52.2) | 9 (450) |  |  |  |
|  | moderate | | 28(15.6) | - |  |  |  |
|  | severe | | 58 (32.2) | 11 (55) |  |  |  |
| YFAS 2.0 |  | |  |  |  | 1.332 | ns |
|  | none | | 111 (61.7) | 13 (65.0) |  |  |  |
|  | mild | | 9 (5.0) | - |  |  |  |
|  | moderate | | 23 (12.8) | 2 (10.0) |  |  |  |
|  | severe | | 37 (20.6) | 5 (25.0) |  |  |  |
|  | | |  |  |  |  |  |
| FPG^†a^ | | | 89.2 (10.4) | 95.1 (11.7) |  | 3.122 | ns |
| FPI^†b^ | | | 18.6 (8.5) | 18.9 (8.9) |  | 0.034 | ns |
| Matsuda index^†c^ | | | 2.7 (1.5) | 2.6 (1.1) |  | 0.095 | ns |
| HOMA-IR^†d^ | | | 4.1 (1.9) | 4.5 (2.4) |  | 0.174 | ns |
| logIGI^†e^ | | | 1.4 (0.3) | 1.5 (0.5) |  | 1.098 | ns |
| GTP^‡^ | | |  |  |  | 9.603 | .022 |
|  | | NGT | 120 (66.7) | 10 (50.0) |  |  |  |
|  | | IFG | 7 (3.9) | 3 (15.0) |  |  |  |
|  | | IGT | 35 (19.4) | 2 (10.0) |  |  |  |
|  | | IFG+IGT | 18 (10.0) | 5 (25.0) |  |  |  |
| *Abbreviations*: BED: binge eating disorder; FA: food addiction; BMI: body mass index; BES: binge eating scale; YFAS: yale food addiction scale; FPG: fasting plasma glucose; FPI: fasting plasma insulin; HOMA-IR: homeostasis model assessment insulin resistance; IGI: insulinogenic index; GTP: glucose tolerance phenotype; NGT: normal glucose tolerance; IFG: impaired fasting glucose; IGT: impaired glucose tolerance.  Insulinogenic index (IGI) was log-transformed before analysis. ^†^ Means and standard deviations; ^‡^ Frequencies and percentages. Model adjusted for diagnosis, BMI, sex, and age, with Bonferroni correction for multiple comparisons set at p<.01.  ^a^ significant effect of age (F=10.383; p=.002; ŋ^2^=0.074).  ^b^ significant effect of BMI (F=19.581; p=<.001; ŋ^2^=0.132), age (F=14.239; p<0.001; ŋ^2^=0.099).  ^c^ significant effect of BMI (F=17.329; p=<.001; ŋ^2^=0.118).  ^d^ significant effect of BMI (F=17.440; p<.001; ŋ^2^=0.119), age (F=6.884; p=.01; ŋ^2^=0.051).  ^e^ significant effect of age (F=18.076; p<.001; ŋ^2^=0.123). | | | | | | | |
